# Transcriptomic analysis of mouse cochleae suffering from gentamicin damage reveals the signalling pathways involved in hair cell regeneration

**DOI:** 10.1038/s41598-019-47051-5

**Published:** 2019-07-19

**Authors:** Huanju Bai, Lingling Jiang, Xi Wang, Xue Gao, Jie Bing, Chao Xi, Weiqian Wang, Meiguang Zhang, Xinwen Zhang, Zhongming Han, Jincao Xu, Shaoju Zeng

**Affiliations:** 1 Beijing Key Laboratory of Gene Resource and Molecular Development, Beijing Normal University, Beijing, 100875 China; 2 Department of Otorhino, Hospital of the PLA Rocket Force, Beijing, 100088 China; 3 Ministry of Education Key Laboratory for Ecology of Tropical Islands, College of Life Sciences, Hainan Normal University, Haikou, 571158 China; 4 Department of Otorhinolaryngolgoy, HeBei YanDa Hospital, HeBei, 065201 China

**Keywords:** Hair cell, Neuroscience

## Abstract

There is a strong capacity for hair cell regeneration after damage in the inner ear of non-mammals. However, mammalian hair cells are substantially unable to regenerate. To obtain insights into the mechanism of this difference, we analyzed the transcriptomic changes in the mouse cochleae suffered from gentamicin damage and compared them with those in the chick cochleae suffered from the same damage. The results indicated that 2,230 genes had significantly differential expression between the gentamicin- and saline-treated mouse cochleae. Some of the differentially expressed genes were grouped into 265 signaling pathways, including the Notch, Wnt (Wingless and INT-1), Bmp (bone morphogenetic protein), FGF (fibroblast growth factor) and Shh (sonic hedgehog) pathways. Using pharmacological inhibitors or agonists of these pathways, the effects of these pathways on hair cell regeneration were further studied. The results indicated that Bmp alone and its coregulation with the Notch or Wnt signaling pathways increased the numbers of generated cells from transdifferentiation or proliferation in the mouse cochlea after damage, in addition to the reported coregulation of Notch and Wnt. Thus, this work indicates a new signaling pathway (Bmp) and its synergetic coregulation in mammalian hair cell regeneration, providing potential therapeutic targets to increase mammalian hair cell regeneration.

## Introduction

The vertebrate inner ear is a sensory organ of exquisite design and sensitivity with a highly ordered and complex architecture^1^. Profound hearing loss and balance disorders are the most common sensory diseases that arise from the degeneration and death of sensory hair cells^2^. Although mammalian hair cell loss in the adult cochlea is permanent, the inner ear has a strong ability to regenerate hair cells in other vertebrates, such as birds and fish^3,4^. This finding raises an intriguing issue: can we use the mechanisms by which hair cells are regenerated in nonmammalian cochleae to induce hair cell regeneration in mammals?

The inner ear develops from the otic placode, a patch of ectodermal thickening around the edge of the anterior neural plate^5^. After progenitor cells have undergone successive cell proliferation, they are progressively restricted to particular cellular phenotypes (hair or supporting cells)^6,7^. The formation of the inner ear is regulated by a multitude of interacting signaling pathways, including sonic hedgehog (Shh), bone morphogenetic protein (Bmp)^8^, Wingless and INT-1 (Wnt)^9^, fibroblast growth factor (Fgf)^10–14^ and Notch^15,16^ signaling pathways. The genes and signaling pathways which play important roles in the embryonic development of inner ear are highly evolutionarily conserved from fish to mammals^6,7^.

The major processes of hair cell regeneration have been well established in the chick^6,16^. In general, most genes and signaling pathways which have been shown to be involved in the embryonic development of hair cells are also required for hair cell regeneration^6,7^. Given the advantages of high-throughput sequencing, cDNA microarrays, SAGE or RNA-seq have been used to investigate a global gene expression during hair cell regeneration in rats or mice^17–20^ and nonmammals^19,21–26^. Our recent study found that about 800 genes were differentially expressed during chick hair cell regeneration, some of which were grouped into approximately one hundred signaling pathways, including Notch, Wnt, Fgf and Bmp. These pathways have been shown to be concerned with hair cell development^6,7^. However, owing to the limited studies performed and the different methods used, no consensus has been obtained, and it still remains largely unknown for the precise molecular mechanisms by which hair cell regeneration is initiated and proceeds. To date, no studies have focused on the comparison of gene expression between mammals and nonmammals. However, this comparison is necessary, as it may facilitate the identification of the molecular signals that direct cell regeneration, and once identified, the mechanisms may be used to initiate hair cell regeneration in the mammalian cochlea^27,28^, as in the nonmammals^3,4^. In addition, recent reports indicate that following the coregulation of Wnt and Notch signaling, the production of regenerated hair cells in the mouse inner ear substantially increases after damage, in contrast to that following the activation of a single pathway^29–31^. This finding suggests that hair cell regeneration may not be regulated by only one signaling pathway, but by the orchestrated interactions of many genes and signaling pathways^32–34^. Although it is challenging to investigate these interactions, it is worth pursuing, as the concurrent manipulation of pathways may be required to fulfill mammalian hair cell regeneration in the adult cochlea.

To address this issue, we first used RNA-seq to analyze the transcriptomic changes in the mouse cochlea at postnatal 1 day (P1) after gentamicin treatment. The differentially expressed genes were compared with those obtained in the chick cochlea at the corresponding damage time. The results indicated that genes in the Notch, Wnt, Bmp, Fgf and Shh signaling pathways showed large differences between the two species. By using pharmacological inhibitors or agonists of these pathways, we further investigated the effect of these signaling pathways (singly or in a coregulated manner) on hair cell regeneration in the mouse. Our study first indicated that Bmp and its coregulation with Notch or Wnt, similar to that of Notch and Wnt, had a significant effect on the production of regenerated mouse hair cells.

## Results

All the mouse cochleae used in this study were dissected from postnatal day 1 (P1), and for RNA extraction to perform RNA-seq analysis, 250–300 cochleae were combined in each group treated with gentamicin or saline. To study the effects of the Notch, MAPK (Fgf), Wnt, Bmp and Shh pathways on hair cell regeneration, the cochleae were cultured in 24-well plates with pharmaceutical inhibitors or agonists of these pathways. For each group, the cultured cochleae were ranged from 6 to 9 (the sampling number n = 6–9).

### Genes expression in the mouse cochleae suffered from gentamicin damage

**Figure 1:**
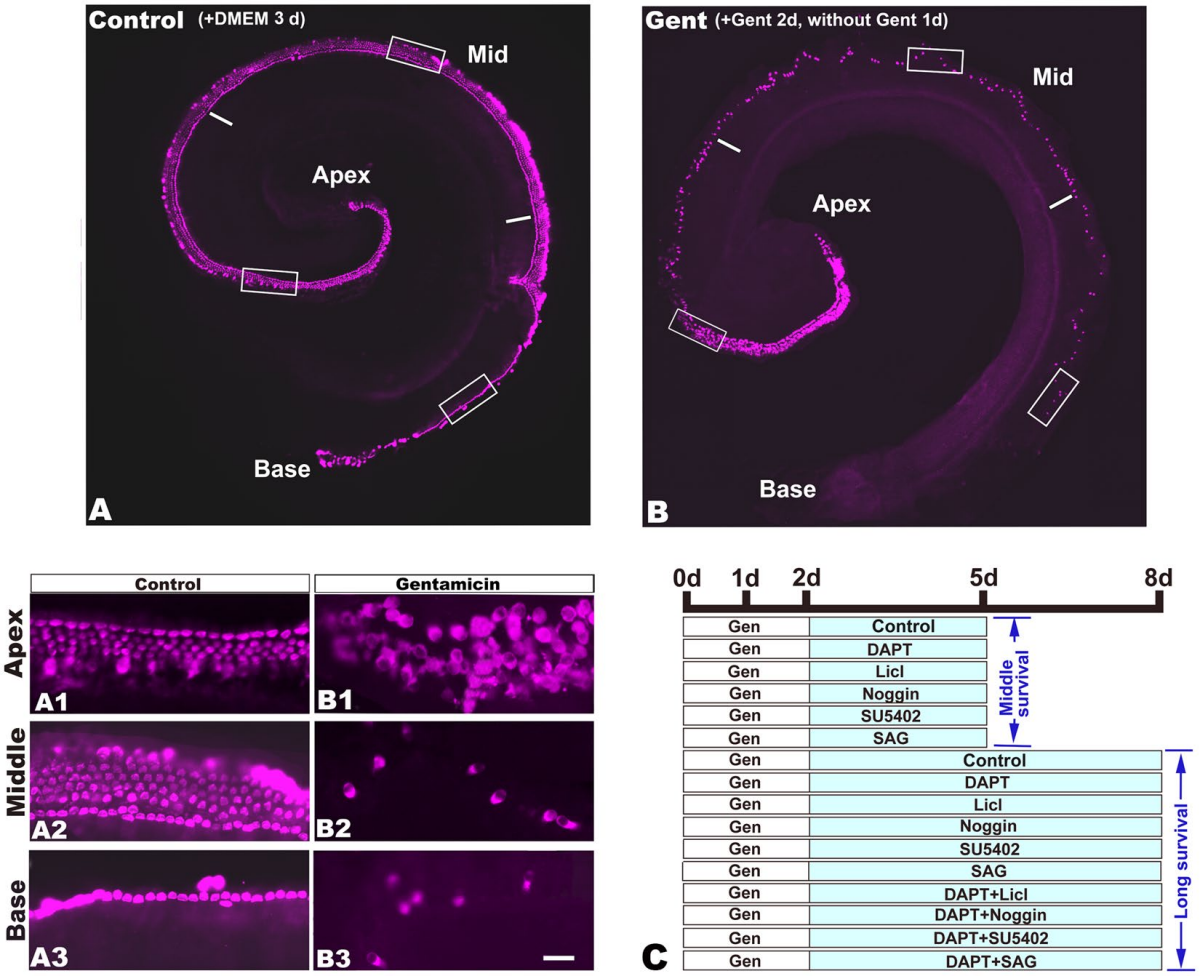
Comparison of hair cells (Myosin 7a+) in mouse Corti organs collected at postnatal day (d) 1 and cultured for 3 d. (**A**) The cochlea cultured in DMEM media. The cochlea is divided into three parts: the apex, middle (mid) and base. A1-A3: The amplified images of the boxed areas in each part of the cochlea in A. Most hair cells in the basal part were lost after 3 d in culture. (**B**) The cochlea cultured in DMEM media with 75 μM gentamicin for 2 d and then without gentamicin for 1 d. B1-B3: The amplified regions are boxed in B. Most hair cells were damaged except the end portion of the apex. (**C**) Schematic for the different treatments of the cultured cochleae after gentamicin (Gen) damage, including the treatments with DAPT (Notch signaling inhibitor), LiCl (Wnt signaling agonist), Noggin (Bmp signaling inhibitor), SU5402 (Fgf signaling inhibitor) or SAG (Shh signaling agonist). The scale bar in B3 = 200 μm for A and B, and 25 μm for A1-B3.

After treatment with gentamicin (0.75 mM) for 2 d and recovery for 1 d, most hair cells in the base and middle parts were injured and lost (Fig. 1A,B). However, most hair cells in the top part of the apex remained intact, while injury became increasingly obvious towards the direction from the apex to the middle. In contrast, hair cells in the middle and apex were intact in the saline-treated control group, and the cellular morphology and cell arrangement remained similar to those isolated freshly dissected. Nevertheless, outer hair cells in most of the base were lost due to mechanical injury (Fig. 1A,B), as previously described^29–31^. As the entire gentamicin-treated or control cochleae were used, the extracted RNAs included those from support cells, hair cells that remained after damage or the cells undergoing regeneration.

**Figure 2:**
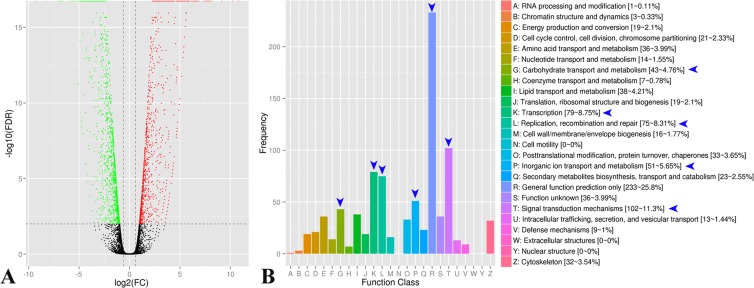
The differentially expressed genes in the neonatal mouse cochleae after gentamicin damage. (**A**) Volcano plots show the differentially expressed genes in the mouse cochleae sampled at postnatal day (d) 1, which were cultured in the medium that contained 75 μM gentamicin for 2 d and collected after 1 d of recovery (without gentamicin in culture medium), in contrast with the control (gentamicin was replaced by the same volume of 0.9% saline). Green and red points indicate the significantly decreased or increased genes, and black points indicate nonsignificantly changed genes. (**B**) Gene enrichment analysis for the differentially expressed genes (fold change ≥1.5) using the COG (Clusters of Orthologous Groups) database. The function groups with large numbers of differentially expressed genes in the cochleae after gentamicin damage are indicated by arrow heads.

As shown in Fig. 2A, 22,086 genes were expressed in the two groups studied (PG3d and the control group, PS3d), of which 2,230 genes were significantly differentially expressed (p < 0.01, fold change ≥1.5) in the gentamicin- or saline-treated cochleae (1,037 upregulated and 1,193 downregulated). These differentially expressed genes are listed in Dataset S1 in which FPKM (fragments per kilobase of exons per million fragments mapped), FDR (false discovery rate), log2FC (fold changes), COG_class and its annotation, Nr (non-redundant) and GO, KEGG, or Swissprot_annotation are shown.

Gene enrichment analysis was further performed for the differentially expressed genes by use of the COG (Clusters of Orthologous Groups) database. The results indicated that the ‘General function prediction only’ (R in Fig. 2B) had the highest frequency with 233 genes differentially expressed (significantly increased or decreased) in the cochleae at 3 d after gentamicin damage, in contrast to the control. The other five top groups of activities were as follows: ‘Signal transduction mechanisms’ (T, n = 102), ‘Transcription’ (K, n = 79), ‘Replication, recombination and repair’ (L, n = 75), ‘Inorganic ion transport and metabolism’ (P, n = 51), and ‘Carbohydrate transport and metabolism’ (G, n = 43) (Fig. 2B).

**Figure 3:**
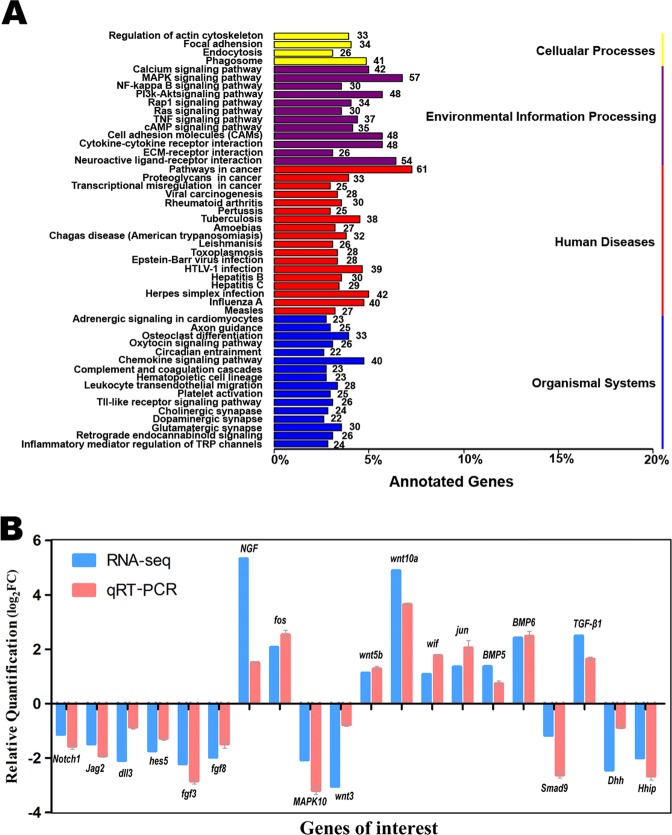
Top 50 metabolic pathways with large numbers of differentially expressed genes in the cochleae after gentamicin damage and the validation of expression changes in RNA-seq analysis with qRT-PCR. (**A**) The top 50 groups of metabolic pathways (on the Y-axis) and the percentage of significantly changed genes in a pathway of the total number of the genes significantly changed after gentamicin damage (on the X-axis). Arabic numerals adjacent to the bars indicate the number of genes differentially expressed after gentamicin damage in each pathway. The groups are further classified into four large classes marked with different colors. (**B**) Comparison of the relative expression levels of the 20 differentially expressed genes in the Notch, MAPK (Fgf), Wnt, TGF-β (Bmp) and Shh pathways (2–5 genes for each pathway) between RNA sequencing (RNA-seq, in blue) and quantitative real-time PCR (qRT-PCR, in shallow red) analyses. The normalized expression level of an examined gene relative to *β-actin* was calculated using the 2^−ΔΔCt^ method, and the expression values are presented as the log_2_fold change (FC) over any of the assigned samples examined (assigned expression value = 1.0).

By use of the KEGG (Kyoto Encyclopedia of Genes and Genomes) database, some genes expressed differentially were grouped into 265 signaling pathways, including the Notch, MAPK, Wnt, TGF-β (Bmp) and Shh pathways, which play important roles in the cochlear development and were also altered in the chicken cochlea suffered from gentamicin damage^6,7,35^. However, these signaling pathways, with the exception of MAPK (Fgf), were not included in the list of top 50 (Fig. 3A), as in the chick^35^, the more details are shown in Table 1 (including the results from the comparison between the mouse and chick). The differentially expressed genes in Notch, MAPK, Wnt, TGF-β, Shh, Cell cycle and Jak-Stat signaling pathways are listed in S Table 1 in the mouse cochleae suffered from gentamicin damage, compared to the control. The original data for RNA-seq analysis have been deposited in the NCBI SRA (www.ncbi.nlm.nih.gov/sra) or GEO (www.ncbi.nlm.nih.gov/gds): SRR8873471 or GSM3720808 (for the group treated with saline) and SRR8873474 or SRR3720809 (for the group treated with gentamicin).

**Table 1:** Comparison of signaling pathways and the genes in each pathway between the mouse and chick (in parentheses) cochleae.

Signaling Pathway	Top 50	Numbers of genes down(↓) or up (↑)-regulated in each pathway
MAPK	Yes	24↑/24↓
	(Yes)	(4↑/12↓)
Notch	No	2↑/2↓
	(Yes)	(2↑/7↓)
Wnt	No	8↑/11↓
	(Yes)	(3↑/4↓)
TGF-β	No	1↑/2↓
	(Yes)	(8↑/3↓)
Shh	No	−1↑/1
	(No)	(2↑/3↓)

### Confirmation of gene expression obtained from RNA-seq analysis

To confirm the gene expression obtained from RNA-seq, we chose 20 genes which were shown to be expressed differentially in the Notch, MAPK (Fgf), Wnt, TGF-β (Bmp) and Shh pathways to be further verified by qRT-PCR (2–5 genes were selected from each pathway). The selected genes included *notch1*, *jag2*, *delta-like* 3 and *hes5* (Notch pathway); *fgf3*, *fgfr3*, and *NGF* (nerve growth factor); *c-fos* and *MAPK10* (mitogen-activated protein kinase 10) (MAPK/Fgf pathway); *wnt3*, *wnt5b*, *wnt10a*, *wif*, and *jun* (Wnt pathway); *Bmp5*, *Bmp6*, *Smad9* and *TGF-β1* (TGF-β/Bmp pathway); and *dhh* and *hhip* (Shh pathway). The primers of these genes are shown in Table 2. The qRT-PCR results were largely consistent with those from RNA-seq (Fig. 3B, n = 3 for each group. RNA in each sample was extracted from 16–20 cochleae).

**Table 2:** Primers for qRT-PCR.

Gene	5′ primer	3′ primer
*Notch1*	GGAGGACCTCATCAACTCACA	CGTTCTTCAGGAGCACAACA
*Jag2*	CGTCGTCATTCCCTTTCAGTT	CTCAATCAGCAGCTCCTCATCT
*DLL3*	GGGGATTCTACGGGCTTCG	CAGGATCTTCACCGCCAACAC
*Hes5*	CCGGTGGTGGAGAAGATGCG	AGCTTGGAGTTGGGCTGGTG
*FGF3*	TGCGCTACCAAGTACCACC	CACCGCAGTAATCTCCAGGAT
*FGF8*	GCCTCCAAGCCCAGGTAAG	GAAGGGTCGGTCCTCGTGT
*NGF*	CCCACCCAGTCTTCCACAT	GTCACCTCCTTGCCCTTGA
*FOS*	CGGCATCATCTAGGCCCAG	TCTGCTGCATAGAAGGAACCG
*MAPK10*	TCAGGGAATAGTCTGTGCTGC	CTCTTGGCGTGAGTTTGGTTC
*Wnt3*	GCCCGCTCAGCTATGAACAA	CCGTGGCATTTACACTTTAGGT
*Wnt5b*	TCCTGGTGGTCACTAGCTCTG	TGCTCCTGATACAACTGACACA
*Wnt10a*	CACTCCGACCTGGTCTACTTTGA	GGGACCCGTGCTGCTCTTAT
*Wif*	ATCCAACTGTCAATGTCCCTT	CACTTCAAATGCTGCTACCC
*Jun*	GGCACATCACCACTACACC	TGTTCTGGCTATGCAGTTCAG
*BMP5*	GGGAGACAATCACGTTCACTC	GAGGCAAACCCAAAATAGACAGA
*BMP6*	GTTGGCTGGAATTTGACATCAC	TCCATCCCGAGTCACCACA
*Smad9*	CACCGACCCTTCCAATAACA	ACACCCTTTCCAATGTGCC
*TGF-β1*	CCACCTGCAAGACCATCGAC	CTGGCGAGCCTTAGTTTGGAC
*Dhh*	GGACCTCGTACCCAACTACAA	CGATGGCTAGAGCGTTCACC
*Hhip*	CAGGTCTTCTTCAAACAACTGCT	TGCTTTCTCGGGAAGTCTGGA
*actin*	TTGGCAATGAGAGGTTCAGGT	TACGGATGTCCACATCACACT

### Effects of the Notch, MAPK (Fgf), Wnt, Bmp and Shh pathways on hair cell regeneration

As indicated in the previous analysis, some genes were expressed differentially in the Notch, Wnt, Bmp, MAPK (Fgf) and Shh signaling pathways in the mouse cochlea. However, most of these signaling pathways were not included in the list of top 50 as in the chick^35^, which suggests that the inability of mammalian hair cell regeneration might be related to the inadequate activation/inactivation of these signaling pathways which are involved in the development of embryonic hair cells^6,7^. We thus investigated the effect of these signaling pathways on mouse hair cell regeneration. In each of the studied groups, the cochlea was divided into three parts, the base, middle and apex (Fig. 1A,B), and the following cells in each part of the cochlea were examined and compared among the studied groups: the cells labeled for Myosin7a+(mature hair cells), Sox2+(supporting cells), Sox2+/BrdU+(mitotic supporting cells), Myosin7a+/Sox2+(hair cells from transdifferentiation), and Myosin7a+/Sox2+/BrdU+(hair cells from mitotic regeneration of supporting cells). A schematic of the studies is shown in Fig. 1C.

### Effect of DAPT (Notch inhibitor) on hair cell regeneration

**Figure 4:**
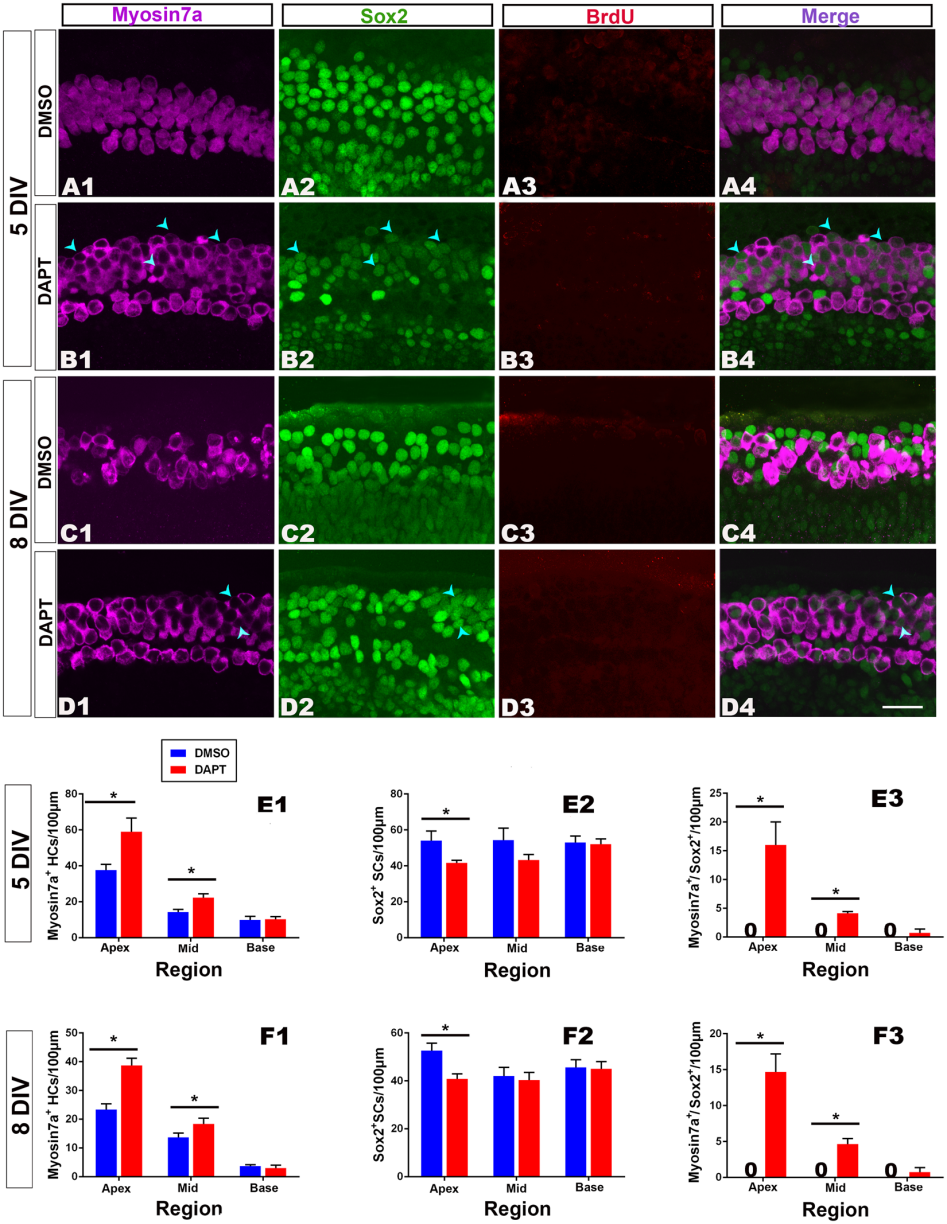
Notch signaling inhibition increases the transdifferentiation of supporting cells with no effect on proliferation. (**A1–D4**) Confocal slices from the apex of neonatal organ of Corti explants. The cochleae were treated with 0.25% DMSO for 3 (**A1–A4**) or 6 (**C1–C4**) days and cultured *in vitro* for 5 or 8 days (5 or 8 DIV). Samples of double-labeled cells for Myosin 7a+(purple)/Sox2+(green) are indicated by blue arrow heads. Cells labeled for BrdU+(red) were lacking in both studied groups (**A3–D3**). The scale bar in D4 is 25 μm for (**A1**–**D4**). (**E1** and **F1**) The number of Myosin 7a+cells per 100 μm increased in the apex and middle parts of DAPT-treated cochleae in contrast to the DMSO-treated cochleae. (**E2** and **F2**) There was a significant decrease in the number of Sox2+cells in the apex (but not middle or base) of DAPT-treated cochleae. (**E3** and **F3**) A significant increase in the number of cells double-labeled for Myosin 7a+/Sox2+in the apex and middle (but not the base) of DAPT treated cochleae. The bar legend for (**E1–F3**) is shown in (**E1**). Data are presented as the mean ± SEM per 100 μm; *p < 0.05, unpaired Student’s *t*-tests (two-tailed).

Following a 3 d (culture *in vitro* for 5d, 5DIV) or 6 d (culture *in vitro* for 8d, 8DIV) treatment with DAPT (50 μM) (n = 6–8 per group), which inhibits the activity of gamma secretase^16^, the density (the numbers of examined cells per 100 μm) of hair cells (Myosin 7a+) indicated significant increases in the apical (5 DIV: t = 4.57, p = 0.013; 8 DIV: t = 4.11, p = 0.028) and middle (5 DIV: t = 4.33, p = 0.018; 8 DIV: t = 3.45, p = 0.031) (but not basal) parts of the cochlea (Fig. 4A1–D1,E1,F1). In contrast, the density of the supporting cells (Sox2+) after treatment with DAPT indicated significant decreases in the apical (5 DIV: t = 2.33, p = 0.038; 8 DIV: t = 3.15, p = 0.034) but not middle and basal parts of the cochlea (Fig. 4A2–D2,E2,F2). Along the whole cochlea, Myosin 7a+/Sox2+cells were observed, and there was a significantly larger number in the apical (5 DIV: t = 12.34, p = 0.001; 8 DIV: t = 13.65, p = 0.004) and middle (5 DIV: t = 9.35, p = 0.003; 8 DIV: t = 9.17, p = 0.002) (but not basal) parts of the cochlea in the DAPT-treated group than in the DMSO-treated group (Fig. 4A1–D4, E3,F3). Few labeled cells for Sox2+/BrdU+were identified in the DAPT treated groups (3.5–4.7 per 100 μm) in the apical part of the cochlea, and no Sox2+/BrdU+cells were identified in the middle and basal parts of the cochlea (Fig. 4A3–D3). Almost no Myosin 7a+/Sox2+/BrdU+cells were observed along the whole cochlea in all studied groups. These data indicate that Notch signaling inhibition has no significant effect on the promotion of mitotic regeneration, while it increases hair cell regeneration from transdifferentiation at the cost of a decrease in the number of supporting cells.

### Effect of LiCl (Wnt agonist) on hair cell regeneration

**Figure 5:**
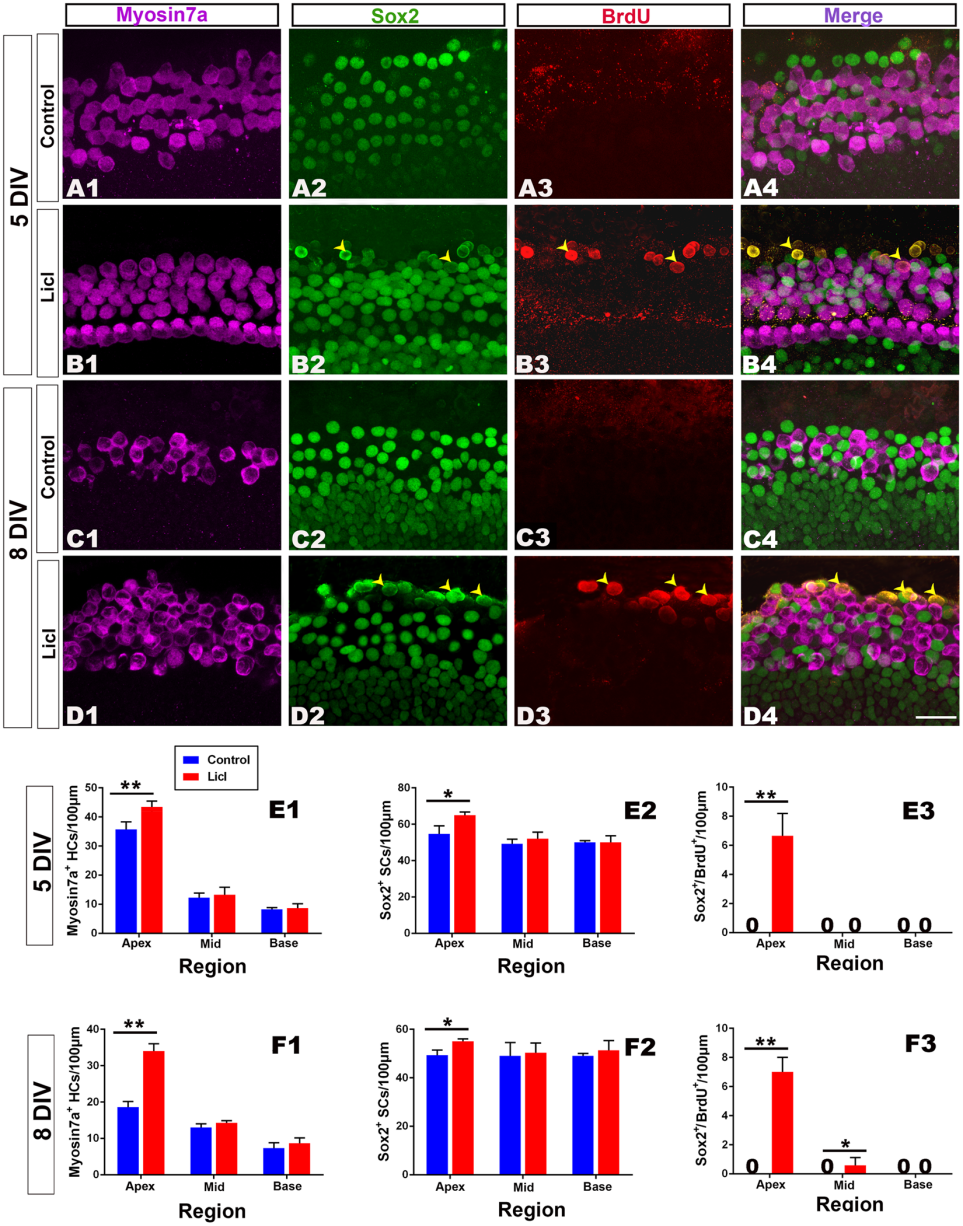
Wnt/β-catenin signaling pathway activation causes proliferation of supporting cells. (**A1–D4**) Confocal slices from the apex of neonatal organ of Corti explants after gentamicin damage. The cochleae were treated with 0.25% DMSO for 3 (**A1–A4**) or 6 (**C1–C4**) days and cultured *in vitro* for 5 or 8 days (5 or 8 DIV). Some double-labeled cells for Sox2+(green)/BrdU+(red, indicated by arrow heads) but not for Myosin 7a+(purple)/BrdU+or Myosin 7a+(purple)/Sox2+, were observed in the LiCl-treated cochleae (**B4** and **D4**). In contrast, cells labeled for BrdU+were lacking in the DMSO treated cochleae (**A4** and **C4**). The scale bar in D4 is 25 μm for (**A1–D4**). The scale bar is 20 μm. (**E1–F3**) The number of examined cells per 100 μm in the sensory region from the apex to the base of the cochlea, including Myosin 7a+cells in 5 DIV (**E1**) or 8 DIV (**F1**), Sox2+cells in 5 DIV (**E2**) or 8 DIV (**F2**) and Sox2+/BrdU+cells in 5 DIV (**E3**) or 8 DIV (**F3**). The number of Myosin 7a+, Sox2+or Sox2+/BrdU+cells per 100 μm significantly increased in the apex of the cochlea between the LiCl- and DMSO-treated cochleae in 5 DIV (**E1**,**E2**) or 8 DIV (**F1**,**F2**). The bar legend for (**E1**–**F3**) is shown in (**E1**). Data are presented as the mean ± SEM per 100 μm; *p < 0.05, **p < 0.01, unpaired Student’s *t*-tests (two-tailed).

Following treatment with an agonist (LiCl, 7.5 mM) for the Wnt signaling pathway^9,30^, significantly more Myosin 7a+cells were found in the apical part of the cochlea in the 5 d and 8 d survival groups (n = 6–9 per group; 5 DIV: t = 4.32, p = 0.028; 8 DIV: t = 8.76, p = 0.011; Fig. 51–D1,E1,F1). In addition, the number of supporting cells (Sox2+) significantly increased in the apical (5 DIV: t = 3.32, p = 0.038; 8 DIV: t = 2.78, p = 0.035; but not middle and basal) part of the cochlea (Fig. 5A2–D2,E2,F2). Some labeled cells for BrdU+or BrdU+/Sox2+(but nearly no Myosin 7a+/BrdU+or Myosin 7a+/Sox2) were identified along the region in which outer hair cells were located in the apical part of the cochlea after treatment with LiCl; however, they were not identified in the control group. Labeled cells for BrdU+/Sox2+were scarce in the middle part of the cochlea and nearly lacking in the basal part of the cochlea in the 5 d or 8 d survival groups (Fig. 5E3,F3). These data suggest that Wnt signaling activation causes the proliferation of supporting cells (BrdU+/Sox2+), while it fails to successfully induce the proliferated cells to differentiate into hair cells (almost no labeled cells for Myosin 7a+/BrdU+/Sox2+). In addition, Wnt signaling activation does not cause hair cell regeneration from transdifferentiation (no labeled cells for Myosin 7a+/Sox2+).

It is needed to note that the staining of Sox2+/BrdU+cells is different from Sox2+/BrdU−. As shown in Figure 5B2,D2, Sox2 staining is usually localized to the nucleus in Sox2+/BrdU- cells. However, the staining highlighted by the yellow arrow heads in Sox2+/BrdU+cells is much more diffuse and seems higher in the cytoplasm and membrane. This might be caused by the decreased staining for Sox2 in supporting cells undertaking cellular proliferation in contrast to the supporting cells without performing proliferation. Thus, the above staining may indicate the varied state of supporting cells.

### Effect of Noggin (Bmp inhibitor) on hair cell regeneration

**Figure 6:**
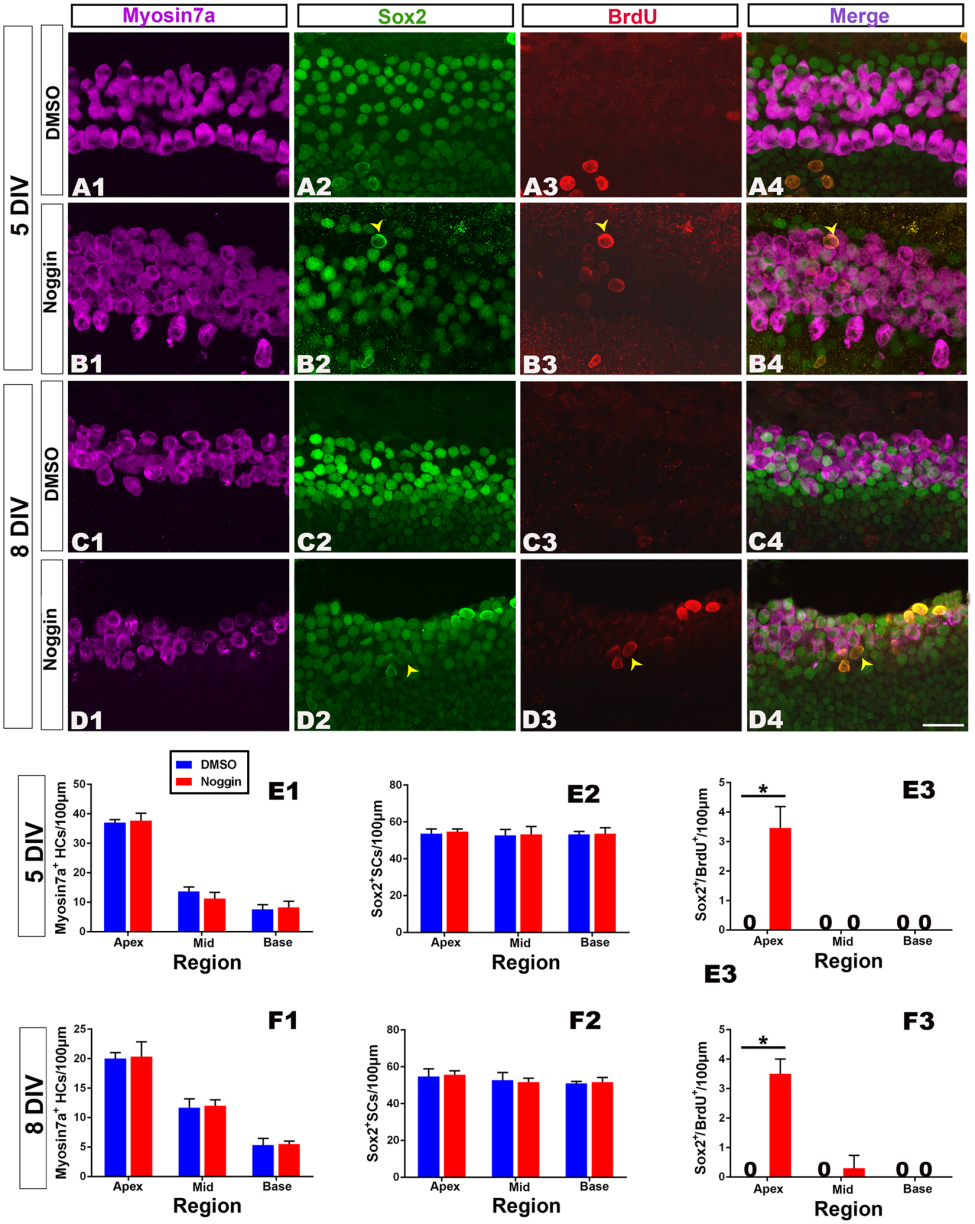
Bmp signaling pathway inhibition causes proliferation of supporting cells. (**A1–D4**) Confocal slices from the apex of the neonatal organ of Corti explants after gentamicin damage. The cochleae were treated with 0.25% DMSO for 3 (**A1**–**A4**) or 6 (**C1–C4**) days and cultured *in vitro* for 5 or 8 days (5 or 8 DIV). Cells double-labeled for Sox2+(green)/BrdU+(indicated by arrow heads), but not for Myosin 7a+(purple)/BrdU+or Myosin 7a+(purple)/Sox2+, were observed in the Noggin-treated cochleae (**B4** and **D4**). In contrast, cells labeled for BrdU+were lacking in the DMSO-treated cochleae (**A4** and **C4**). The scale bar in D4 is 25 μm for (**A1**–**D4)**. It is needed to note that some BrdU+/Sox2+cells in the DSMO control shown in (**A3**) were located outside of the region where inner or outer hair cells were located. Thus, they were not counted and compared. (**E1**–**F3**) The number of Myosin 7a+, Sox2+or Sox2+cells per 100 μm in the sensory region from the apex to the base of the cochlea did not significantly change between the Noggin- and DMSO-treated cochleae in 5 DIV (**E1**,**E2**) or 8 DIV (**F1,F2**). There was a significant increase in the number of Sox2+/BrdU+cells per 100 μm in the apex of cochleae in the Noggin-treated group, in contrast to that in the DMSO-treated group (**E3**: 5 DIV; **F3**: 8 DIV). The bar legend for (**E1–F3**) is shown in (**E1**). Data are presented as the mean ± SEM per 100 μm; *p < 0.05, **p < 0.01 unpaired Student’s *t*-tests (two-tailed).

After culture with Noggin (0.16 μg/mL), an inhibitor of Bmp signaling^36,37^, the number of hair cells (Myosin 7a+) or supporting cells (Sox2+) per 100 μm did not show significant changes along the whole cochlea in the 5 d or 8 d survival groups (Fig. 6A1–D2,E1,F2). However, Sox2+/BrdU+cells were identified along the region in which outer hair cells were located. These Sox2+/BrdU+cells were mainly located in the apical part of the cochlea (Fig. 6B3,D3), and some cells were in the middle part, with none in the basal part. The numbers of Sox2+/BrdU+cells per 100 μm significantly increased in the apical (n = 6–9 per group; 5 DIV: t = 14.57, p = 0.001; 8 DIV: t = 13.48, p = 0.001) part of the cochlea after treatment with Noggin in the 5 d survival group (Fig. 6A3–D4,E3,F3); however, they did not significantly change for the other examined groups. These data indicate that Bmp signaling inhibition promotes the proliferation of supporting cells (Sox2+/BrdU+). No labeled cells were observed for Myosin 7a+/BrdU+/Sox2+or Myosin 7a+/Sox2+in the Noggin-treated group, which suggests no hair cell regeneration from proliferation or transdifferentiation.

### Effect of SU5402 (Fgf2 inhibitor) or SAG (Shh signaling agonist) on hair cell regeneration

**Figure 7:**
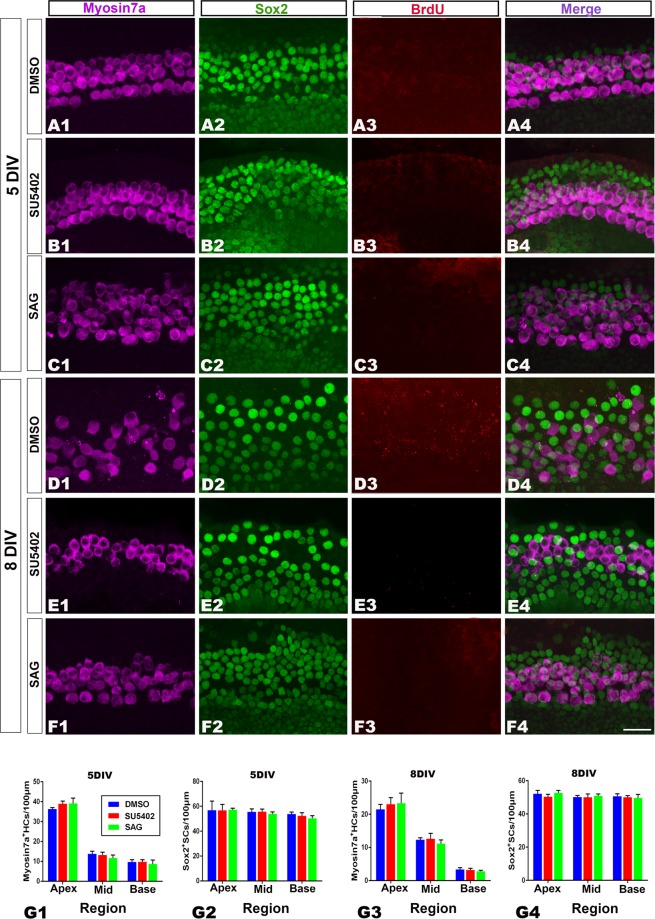
Fgf signaling pathway inhibition and Hedgehog signaling activation had no effect on cell proliferation and transdifferentiation. (**A1–F4**) Confocal slices from the apex of neonatal organ of Corti explants after gentamicin damage. The cochleae were treated with 0.25% DMSO for 3 (**A1**–**A4**) or 6 (**C1**–**C4**) days and cultured *in vitro* for 5 or 8 days (5 or 8 DIV). No BrdU (red)-labeled cells or double-labeled cells for Myosin 7a+(purple)/Sox2+(green) were observed in the studied groups. The scale bar in F4 is 25 μm for (**A1–F4**). (**G1–G3**) The number of Myosin 7a+or Sox2+cells per 100 μm in the sensory region from the apex to the base of cochlea did not significantly change among the studied groups. The bar legend for (**G1–G4**) is shown in (**G1**). Data are presented as the mean ± SEM per 100 μm; unpaired Student’s *t*-tests (two-tailed).

After the addition of SU5402 or SAG, the number of Myosin 7a+or Sox2+cells did not significantly change compared with the control groups (n = 6–9 per group) (Fig. 7). None of the examined cell types, including Sox2+/BrdU+, Myosin 7a+/BrdU+/Sox2+and Myosin 7a+/Sox2+, were found along the whole cochlea (Fig. 7).

### Coregulation between DAPT and Noggin, LiCl, SU5402 or SAG on hair cell regeneration

**Figure 8:**
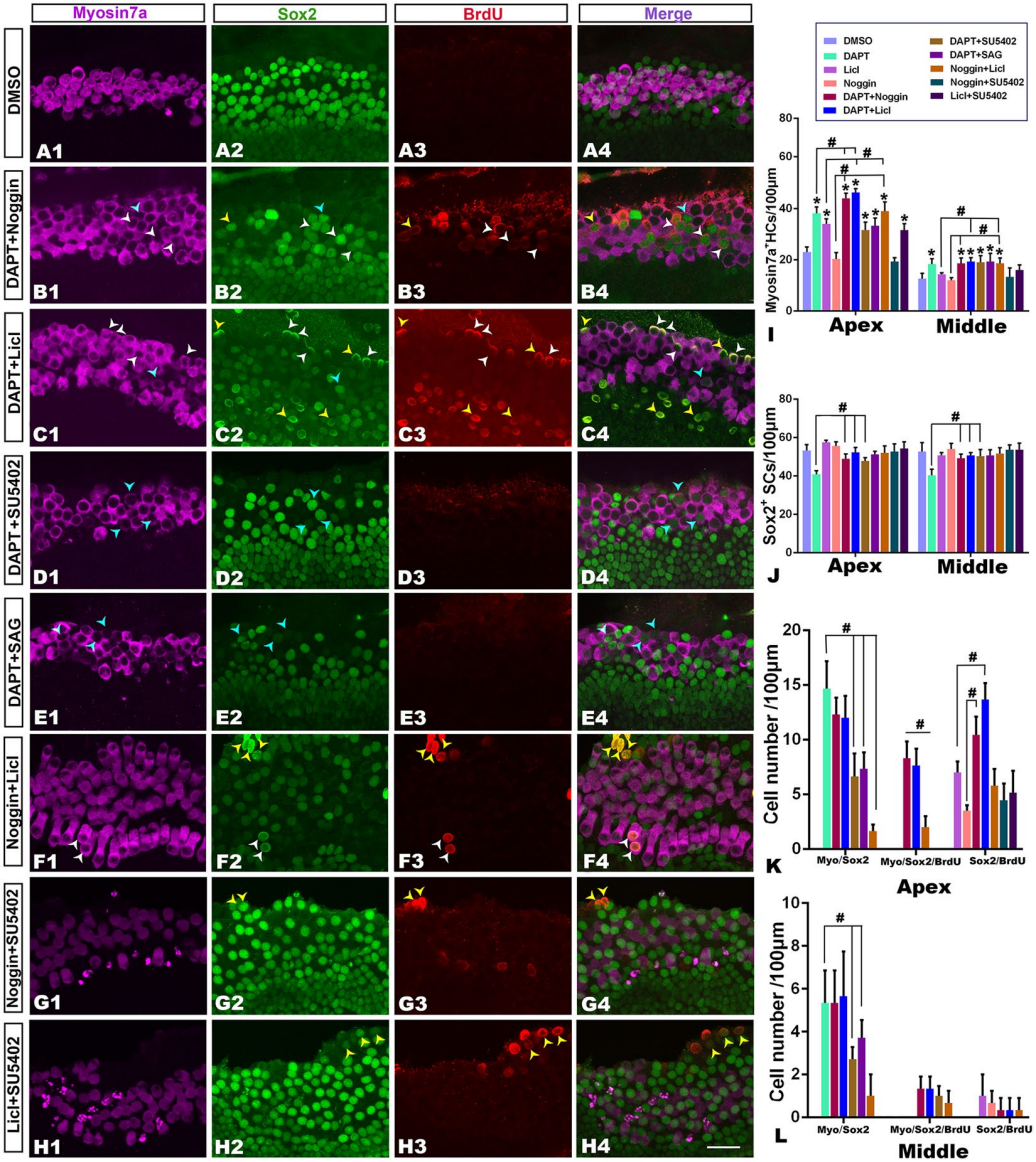
Coregulation of Notch and Wnt, Bmp4, Fgf or hedgehog signaling pathways on hair cell regeneration. (**A1–H4**) Confocal slices from the apex of the neonatal organ of Corti explants after gentamicin damage. The cochleae were treated with 0.25% DMSO (**A1–A4**) DAPT + Noggin (**B1–B4**), +LiCl (**C1–C4**), +SU5402 (**D1**–**D4**) or +SAG (**E1–E4**), Noggin + LiCl (**F1–F4**) or +SU5402 (**G1–G4**), and LiCl + SU5402 (**H1–H4**) for 6d. The labeled cells for Myosin 7a+(purple)/Sox2+(green) are indicated by blue arrow heads, the labeled cells for Sox2+/BrdU+(red) are indicated by yellow arrow heads, and the labeled cells for Myosin 7a+/Sox2+/BrdU+are indicated by white arrow heads. The scale bar in H4 is 25 μm for (**A1–H4**). (**I** and **J**) The comparison of the number of Myosin 7a+(**I**), Sox2+(**J**) cells per 100 μm in the sensory region in the apex and middle of the cochleae among the studied groups. (**K** and **L**) The comparison of the number of labeled cells for Myosin 7a+/Sox2+, Myosin 7a+/Sox2+/BrdU+, and Sox2+/BrdU+cells per 100 μm in the apex (**K**) and middle (**L**) parts of the cochleae in the studied groups. The bar legend for I-L is shown in (**I**). “*” in I indicates significant differences in contrast to the control (DMSO) (p < 0.05). “#” in I–L indicates significant differences between the compared groups (p < 0.05). Data are presented as the mean ± SEM per 100 μm; One-way ANOVA with Tukey’s post hoc tests.

Finally, we determined whether there is coregulation among these signaling pathways. As shown in Fig. 8, hair cell regeneration from transdifferentiation (Myosin 7a+/Sox2+, marked with blue arrowheads) or proliferation (Myosin 7a+/BrdU+/Sox2+, marked with white arrowheads) were identified in three coregulated groups cultured *in vitro* for 8d (8DIV): DAPT & Noggin (Fig. 8B1–B4), DAPT & LiCl (Fig. 8C1–C4) or LiCl & Noggin (Fig. 8D1–D4). In contrast, only transdifferentiated cells (Myosin 7a+/BrdU−/Sox2+) were identified in the group treated alone with DAPT (Fig. 4), and the proliferated support cells (Myosin 7a−/BrdU+/Sox2+) were found in the groups treated with LiCl (Fig. 5) or Noggin (Fig. 6) alone.

We counted and compared the numbers of hair cells (Myosin 7a+) and support cells (Sox2+) among the studied groups (n = 6–9 per group). As most generated cells were located in the apex and middle of the cochlea, the analysis was not performed in the base of the cochlea. Statistical analyses indicated that Myosin 7a+hair cells significantly changed among the studied groups in the apex (F = 42.33, p < 0.001) or middle (F = 5.13, p = 0.001). Compared to the DMSO control group, Myosin 7a+hair cells significantly increased in the apex of the cochlea in all coregulated groups with the exception of the group treated with Noggin & SU5402 (Fig. 8I). In addition, Myosin 7a+hair cells showed significant increases in the groups treated with DAPT & Noggin, DAPT & LiCl or Noggin & LiCl, compared with the group treated alone with DAPT, Noggin or LiCl (Fig. 8I). In the middle of the cochlea, compared with the group treated alone with Noggin or LiCl, Myosin 7a+hair cells significantly increased in the groups treated with DAPT & Noggin or DAPT & LiCl (Fig. 8I). These data indicated that the coregulation of Notch, Wnt and Bmp signaling pathways had significant synergetic enhancement in the increase of the number of Myosin 7a+hair cells.

The numbers of Sox2+/BrdU+cells per 100 μm changed significantly among the studied groups in the apex (F = 3.57, p = 0.006) or middle (F = 4.19, p = 0.003) (Fig. 8J). In the apex or the middle of the cochlea, compared with the group treated alone with DAPT, support cells (Sox2+) significantly increased in the groups treated with DAPT & Noggin or DAPT & LiCl (Fig. 8J). These data indicated that the loss of support cells was prevented from the coregulation of DAPT with Noggin or LiCl by the proliferation of supporting cells, in contrast to single treatment with DAPT in which the proliferation is almost lacked (Fig. 4F2).

The numbers of regenerated cells from transdifferentiation (Myosin 7a+/Sox2+) or the proliferated support cells (Sox2+/BrdU+) per 100 μm changed significantly among the studied groups (p < 0.001). The numbers of regenerated cells from transdifferentiation (Myosin 7a+/Sox2+) in the apex or the middle of the cochlea, which appeared in the coregulated groups of DAPT & Noggin or DAPT & LiCl (but not DAPT & SU5402, DAPT & SAG or Noggin & LiCl) reached a similar level to that in the group treated alone with DAPT (p > 0.05; Fig. 8K,L). Compared with the group treated alone with Noggin or LiCl, the proliferated support cells (Sox2+/BrdU+) significantly increased in the apex of the cochlea in the coregulated groups with DAPT & Noggin or DAPT & LiCl (Fig. 8K), which indicates that the proliferation of support cells was enhanced after the addition of DAPT with Noggin or LiCl. However, the proliferated support cells (Sox2+/BrdU+) did not show significant changes in the groups treated with Noggin & SU5402, Noggin & LiCl or LiCl & SU5402, compared with the groups treated alone with Noggin or LiCl (Fig. 8K).

## Discussion

Our high-throughput transcriptomic analysis indicated that some genes in the Notch, Wnt, TGF-β (Bmp), MAPK (Fgf) and Shh signaling pathways were differentially expressed in gentamicin treated mouse cochleae. However, they were not included in the top 50 signaling pathways as in the chick^35^. Regarding the roles of these signaling pathways in embryonic production^6,7^ and the damage-induced hair cell regeneration in the chick^35^, we further investigated whether these signaling pathways were concerned with mouse hair cell regeneration. Our results indicated that, in addition to the Notch and Wnt signaling pathways, Bmp (but not Fgf and Shh) has a significant effect on mouse hair cell regeneration, and there was an obvious synergetic enhancement in the number of regenerated hair cells after the coregulation of Bmp & Notch or Bmp & LiCl, in addition to the reported Notch & Wnt^29–31,38^.

### Signaling pathways involved in hair cell regeneration were shown by transcriptomic comparison

This study reported a large-scale analysis of changes in gene expression at 3 d after gentamicin addition. According to the previous reports^26,35^, this sample time point corresponds to the time in which the number of genes expressed after gentamicin damage reached a peak during 1 to 7 d after gentamicin treatment in the chick utricle or cochlea. At this time, a large number of supporting cells undergo proliferation, and some new hair cells have been produced by direct phenotypic conversion^16,27,39^. Thus, the genes which play roles in regulating cell proliferation and differentiation (including direct transdifferentiation) should be included in the collected transcriptomic data. To compare the results obtained in the present study with those obtained in the chick cochlea^35^, we used the same aminoglycoside (gentamicin) and the same sampling time.

Our transcriptomic analysis indicated that the number of differentially expressed genes in the mouse cochlea after damage was larger than that previously reported in the chick (2,230 from 22,086 in the mouse vs 805 from 16,588 in the chick), and the differentially expressed genes were grouped into 265 signaling pathways in the mouse cochlea compared with 99 pathways in the chick^35^. It is noted that genes in the Notch, Wnt, Bmp, and MAPK (Fgf) signaling pathways and those involved in cell cycle regulation were all included in the list of top 50 in the chicken cochleae, while most of them were not in the mouse cochleae (Table 1). The reason why hair cells in the mouse cannot be renewed might be ascribed to the fact that these signaling pathways are not adequately activated or inactivated. In addition, much fewer genes (805) were differentially expressed in the chick cochlea after treatment with gentamicin^35^, in contrast to the mouse cochlea (2,230 genes) in the present study. This might be caused by the initiation of hair cell regeneration in the chick cochlea, and it could inhibit the activation of other signaling pathways that are not closely related to hair cell regeneration, resulting in fewer genes expressed differentially.

Although twenty genes differentially expressed in the Notch, MAPK (Fgf), Wnt, TGF-β (Bmp) and Shh pathways were selected for further verification by qRT-PCR, and the qRT-PCR results were largely consistent with the changes in gene expression obtained from RNA-seq, the localization of these genes in the cochlea was not yet known without performing *in situ* hybridization. This is needed to be performed in future.

The total number of genes detected in the present study is largely consistent with a previous report in which approximately 25,000 genes in mouse organogenesis were identified using cDNA microarrays^40^. In addition, several genes in the previously noted signaling pathways were reported in previous studies, including those in Wnt, Fgf and Notch in the chick^41^, and Fgf1/12, Jag1, Hes5, Atoh1 or Sox2 in the mouse^17^. However, due to the different study aims and the methods used, most previous reports on large-scale gene expression did not well correspond to this study in the kinds of genes or the total numbers of genes expressed differentially. For example, by using gene arrays, fewer genes were reported to be expressed differently in the adult rat cochlea suffered from noise treatment^42,43^, but more genes were found to be expressed differentially in the mouse cochlear sensory epithelia in postnatal day-3, compared to those at adult^17^. In general, the RNA presence and quantity can be indicated from a whole genome in time using RNA-seq, whereas cDNA microarrays only identify the limited allele variants designed into the microarrays^20,44^. Thus, this study provides a relatively more complete candidate genes or pathways which play roles in hair cell regeneration in the mouse cochlea, particularly from the comparative perspective with chick hair cell regeneration.

In addition, it is needed to note that both STAR and Tophat2 software are used to align and compare reads of various lengths produced by gene sequencing^45,46^, and no significant differences of results could be produced by using the two methods^45^. Considering that the longer the gene, the greater the number of reads, the RPKM not the gene counts was used to exclude the effect of gene length. The ratios of the FPKM values were adopted to assess the differences in the abundance between the samples. As the same method was used in our recent study on the chick cochleae suffered from gentamicin damage^35^, this is helpful for comparing the present results with those from the chick cochlea, and the expression differences of some genes in several signaling pathways involved in the development of cochlea were found, as discussed above. With the consideration for the above comparison, other normalized measurements including TPM (Transcripts Per Million) were not used in the present study.

### Signaling pathways involved in the damage-induced hair cell regeneration

Our study indicated that the Wnt, Bmp and Notch (but not Fgf and Shh) signaling pathways play roles in damage-induced hair cell regeneration in the mouse cochlea. In addition to the reported coregulation between Notch and Wnt^8,30,31^, Bmp had a significant synergetic enhancement with Notch or Wnt in the increase of the total number of hair cells (Myosin 7a+) and the number of generated cells from transdifferentiation (Myosin+/Sox2+) or proliferation (Myosin+/Sox2+/BrdU+). In addition, the loss of support cells was prevented from the coregulation of DAPT with Noggin or LiCl, in contrast to a single treatment with DAPT. Thus, our study reported a new signaling pathway (Bmp) and its coregulation with Notch or Wnt in damage-induced hair cell regeneration. However, the present study only used the immunohistochemical double staining for myosin VIIA+cells with Sox2 (VIIA+/Sox2+, origin from transdifferentiation) or BrdU+(VIIA+/BrdU+, origin from proliferation) to mark newly differentiated hair cells. More study is needed to address how the generated hair cells were changed from supporting cells in detail by use of cell fate mapping in future.

Although the role of the Bmp signaling pathway is not reported in mammalian hair cell regeneration after damage, it is crucial for the embryonic development of rat and chick sensory epithelium^47,48^. There is a typical temporal and spatial expression pattern of Bmp4 during the embryonic development of hair cells^49,50^. Antagonizing Bmp4 via Noggin can lead to the deformities of the otic capsule^36,37^, whereas exogenous recombinant Bmp4 causes the decreases in the number of hair cells in the vesicle of the chicken ear^48^. It is reported that Bmp activation lies upstream of both Wnt and Fgf^51,52^, and Bmp target genes can be activated independently or cooperatively with Wnt through interaction between Smads and the HMG box domain of Lef1/Tcf^53^. Moreover, synergy between Bmp and Wnt signaling to regulate developmental events has been observed in various cases and may involve direct interactions between Lef1 and Smad proteins^53^. These reports provide support for our results to show a synergetic enhancement of Bmp and Notch or Wnt in hair cell regeneration. However, due to the few studies of Bmp on hair cell regeneration, more studies are needed to address the role of Bmp signaling on hair cell regeneration.

The Wnt signaling pathway indicates a dual function in the developing mouse auditory system by promoting the proliferation of hair cell progenitors that express Sox2 or Lgr5 (leucine-rich repeat-containing, G-protein-coupled receptor, a downstream gene of Wnt)^29,54,55^ and specifying hair cells through the binding of β-catenin to the enhancer region of Atoh1^55,56^. Thus, Wnt can promote the proliferation of hair cell progenitors and specify the differentiation of hair cells^13,29^. However, Wnt activation alone does not produce significant amounts of regenerated hair cells^29–31^. It is reported that Wnt signaling lies upstream of some Bmp or Notch target genes, including Notch1, Hes1 and Jagged1 whose 5′promoter region contains specific Tcf/Lef sites for β-catenin binding^57^. In this study, the number of regenerated hair cells largely increased in the groups treated with LiCl and DAPT or Noggin, compared to those in the group treated with LiCl alone, supporting the synergetic co-regulation of Wnt and Notch or Bmp.

As shown in Results, there is an increase in the number of supporting cells in the groups treated with LiCl, which is matched by the proliferation of supporting cells (Sox2+/BrdU+). There is also an increase in hair cells. However, no proliferation of myosin + cells (myosin+/BrdU+) was seen nor transdifferentiation of myosin + cells (myosin+/sox2+) were observed. The increases of hair cells might result from the decreased loss of hair cells in the LiCl treated group, with reference to a previous report to indicate that β-catenin overexpression inhibited caspase mediated cell apoptosis in the mouse cochlea induced by neomycin injury^58^.

The Notch signaling pathway has important roles in cell fate determination, such as hair cells (Atoh1+) through Notch-targeted genes, including HES family members, cyclin dependent kinase inhibitors (CDKIs, such as p27kip1 or P21) and Myc^29,57,59^. In addition, a recent study indicated that the Notch pathway is greatly attenuated from P0 supporting cells with over 2,000 transcripts to P5 supporting cells with only 20 transcripts significantly altered after treatment with DAPT, and such difference might be a reason for the mammalian cochlea’s inability to regenerate at the end of first postnatal week^19^. In addition, Maass *et al*. (2015) reported that Notch inhibition promoted the transdifferentiation of supporting cells to hair cells within the first postnatal week, which is consistent with our present result^60^. It has been further shown that Notch signaling hinders the proliferation and differentiation of SCs into HCs in localized regions by inhibiting Wnt activity in the zebrafish lateral line^38^, although it is unknown how Notch signaling regulates the activity of the direct Wnt target genes. The present study indicated that regenerated cells were also mainly from trans-differentiation (Myosin7a+/Sox2+), not from cell proliferation (Myosin 7a+/Sox2+/BrdU+) (Fig. 8K). However, the number of regenerated cells from cell proliferation (Myosin 7a+/Sox2+/BrdU+) increased significantly after addition of DAPT with Noggin or LiCl, in contrast to it in the group treated with DAPT alone (Fig. 8K). This might be ascribed to the synergetic interactions among the Notch, Bmp and Wnt.

Fgf signaling pathway is shown to induce the production of the otic placode and the generation of the mosaic structure between hair cells and supporting cells^11,61^, or the chick hair cell regeneration^35,62^. After treatment with SU5402, a dose dependent increase in proliferated hair cells (BrdU+) and support cells (Sox2+) was found in embryonic basilar papillae of chick^55^. However, no significant effects were detected after treatment with SU5402 alone or its co-regulation with DAPT, LiCl or Noggin. The role of Fgf in the mammalian hair cell regeneration is needed to be further clarified.

Although our study provides a relatively more complete candidate genes or pathways which have important roles in damage-induced hair cell regeneration in the mouse cochlea, and indicates some new coregulation of several signaling pathways, the precise molecular mechanisms of these interacted pathways remains unclear. Further efforts are needed to study how these interactions synergistically perform to guide mammalian hair cells to be regenerated fully as in nonmammals.

## Methods

### Animals

Neonatal KM (Kunming) mice were obtained from the Beijing Vital River Laboratory Animal Technology Co., Ltd. All experiments performed in this study were followed the Beijing Laboratory Animal Welfare and Ethics Review guidelines. The Animal Management Committee of the College of Life Sciences, Beijing Normal University approved all the protocols.

### Cochlear explant culture and RNA isolation preparation

After the mice (postnatal day 1, P1) were euthanized by decapitation and the heads were immersed in alcohol for several seconds, they were placed in a glass bottom Petri dish on an ice bag. The cochleae were dissected under aseptic conditions as previously described^63^. The cochleae were then placed in ice-cold sterile hanks buffered saline solution (HBSS; Sigma-Aldrich). The cochleae were cultured in 24-well culture plates coated with a thin layer of Collagen Type I Rat Tail (Corning, 354236). The cochleae were incubated in DMEM (300 μl, Gibco, 11995-065), supplemented with 1% FBS (Gibco, 16000-044), 1% N2 supplement (Gibco, 17502-048), 2% B27 supplement (Gibco, 17504-044), 1% L-Gln (Gibco, 25030081), and 1500 U/mg Penicillin G (Amresco, E480) at 37 °C with 95% air/5% CO_2_. The medium was changed every day. Gentamicin (0.75 mM, Sigma-Aldrich) was used to injure hair cells, and it was added to the culture medium for the first 2 d (the same volume of saline was added for the control), and the cochlear ducts were cultured in the medium without gentamicin for 1 d to allow recovery. To facilitate the comparison of gene expression in the cochlea after aminoglycoside damage between the mouse and chicken, particularly with our recent transcriptomic analysis of chick cochlea after gentamicin injury, this study employed the same sample time (2 d for gentamicin treatment and 1 d recovery) as in the study on the chick^35^.

For both the gentamicin-treated and control groups, the cochleae (n = 250–300 from approximately 150 mice per group) were collected directly into Trizol (GeneCopoeia, America, E01010A), and they were stored at −80 °C for RNA isolation^61^.

Total RNA Kit (TIANGEN, Beijing, DP419) was used to extract total RNA. The sample quality was determined using a formaldehyde agarose gel (no smearing of ribosomal bands was visible), and 260/280 ratios (between 2.0 and 2.1) were calculated using an Ultraspec 3000 spectrophotometer.

### RNA-seq and data analysis

After mRNA in each group was enriched by use of an NEB Next Poly(A) mRNA Magnetic Isolation Module (NEB, E7490), cDNA libraries were constructed, following the manufacturer’s instructions for Illumina mRNA-seq. Briefly, after the enriched mRNA was fragmented, first-strand cDNA was generated by using random primers. Then, Second-strand synthesis, end repair, poly-A tail addition and adaptor ligation were performed. All the RNA-seq libraries constructed were sequenced by using the Illumina HiSeqTM 2500 system. The average correlation coefficient between technical replicates was greater than 0.998.

After the adaptor sequence and low-quality unknown sequences were removed, the clean reads in the FASTQ format were mapped to the *mouse* genome (//ftp.ensembl.org/pub/release-78/fasta/*mus_musculus*/), and compared by using Tophat2 software^46^. Then, the aligned results in the BAM/SAM format were further analyzed with Cufflinks software^64^ and expressed in FPKM (fragments per kilobase of exons per million fragments mapped). The ratios of the FPKM values were used to assess the differences in the abundance between the samples. The differential gene expression in the comparisons was assessed by EBseq^65^. Differentially expressed genes (DEGs) were defined as those with a fold change of ≥1.5 and a false discovery rate (FDR) of <0.01.

Several complementary approaches were used to annotate the DEGs. The sequences were first compared in several protein databases, including the NCBI nonredundant protein (Nr), NCBI nonredundant nucleotide sequence databases (Nt) and Swiss-Prot, with a cut-off E-value of 10^–5^ by BLASTx or BLASTn. For gene annotations, the top hits were retrieved. After the gene sequences were mapped to the COG (Clusters of Orthologous Groups) database, gene functions were predicted and sorted by using Blastall software. The Kyoto Encyclopedia of Genes and Genomes online with Perl scripts was used to sort gene pathways.

### Quantitative real-time PCR

Twenty genes were selected to perform qRT-PCR to confirm the results from the above RNA-seq. The primers for the chosen genes were designed with the Primer3 output program (Table 1). TIANScript RT Kit (TIANGEN, KR104) was used to synthesize the first-strand cDNA. TransStart Green qPCR SuperMix (TransGen Biotech, AQ101-03) was used for qRT-PCR with 10–20 nM cDNA template (2–4 μl) and 0.2 μM each primer (20 μl total volume), which was performed with an ABI 7500 real-time PCR machine (Applied Biosystems). The cycling conditions were 60 °C for 2 min, 95 °C for 10 min, and 40 cycles at 95 °C for 15 s and 60 °C for 1 min. Each sample for qRT-PCR assay was performed in triplicate, and each had a single peak of the melting curve produced at an acquired fluorescence and melting temperature. For each of the examined groups, the expression value of β-actin was used as a normalization control, and the normalized expression level of an examined gene relative to β-actin was calculated by use of the 2^−ΔΔCt^ method.

### NCochlear explant culture and treatment

As previously described, the isolated cochleae from P1 mice were obtained and cultured in DMEM that contained 0.75 mM gentamicin sulfate (Sigma) for 2 d. The cochleae were cultured for an additional 3 d or 6 d (the total time for cochlear culture *in vitro* was 5d or 8d, thus called 5 DIV or 8 DIV groups) in media without gentamicin. During the culture, inhibitors or agonists were added to the culture medium to block or activate the four signaling pathways. The used inhibitors or agonists included 50 μM DAPT (Notch signaling inhibitor, Calbiochem, 565770), 7.5 mM LiCl (Wnt signaling agonist, Sigma), 0.16 μg/mL Noggin (Bmp signaling inhibitor, Sigma-Aldrich, SRP4675), 10 μM SU5402 (FGF signaling inhibitor, Santa Cruz, SC-204308A) and 1 nM SAG (Shh signaling agonist, Selleck, 7779). DMSO (0.25%), the vehicle for the inhibitors or agonists, was used as a control. The chosen doses of the drug were determined in our preliminary experiments to obtain the dose-response curves (at least five drug concentrations were examined) with reference to the protocol of the drugs or previous reports^30,35^, and the doses with the best effect were selected. To label the proliferating cells, 5-bromo-2-deoxyuridine (BrdU, Sigma-Aldrich) was added to the media (1 µM) during the whole culture (5 or 8 d). At least 6 cochleae in each group were simultaneously cultured. During the culture, the medium was changed every day. Using buffered 4% paraformaldehyde, the cochleae after culture were fixed for 4 h at 4 °C.

### Immunohistochemistry and microscopic imaging

The cultured cochleae were processed for immunofluorescence with standard methods. Prior to the immunoreactions, all the cochleae were first treated for 30 min at room temperature with blocking solution (5% normal donkey and bovine serum in 0.05% Triton X-100 in PBS, pH 7.4). The cochleae were then incubated with the primary and secondary antibodies for each 2 h at room temperature. The used primary and secondary antibodies included: rabbit anti-Myosin7a (1:500, Proteus BioSciences, 25–6790), goat anti-Sox2 (1:300, Santa Cruz Biotechnology, sc-17320), rat anti-BrdU (1:2000, ABD Serotec, OBT0030CX), Alexa 647-conjugated donkey anti-rabbit IgG for Myosin7a labeling (cat. 711-606-152), DyLight 488-conjugated bovine anti-goat IgG for Sox2 labeling (cat. 805-545-180), and Alexa 594-conjugated donkey anti-rat IgG for BrdU labeling (cat. 712-585-150). The specificity of the antibodies has been declared in the protocols and proved in our preliminary experiments. All secondary antibodies were obtained from Jackson ImmunoResearch and diluted at 1:400. After the cochleae were sealed with Antifade Solution (Applygen), they were examined under an inverted Zeiss Axio Observer Z1 fluorescence microscope (20×) and an inverted Zeiss LSM700 laser-scanning confocal microscope (40×). Images were obtained using AxioVision 4.8 and Zen Light Edition programs.

### Cell counting and statistical analyses

For cell counting, the stacks with 0.75 μm thickness were combined into an image along the Z-axis. Hair cells (Myosin7a+), supporting cells (Sox2+) and mitotic cells in the apical, middle and basal regions, respectively, were analyzed. Among each part (apex, middle and base) shown in Fig. 1A–C, at least three nonoverlapping fields (220 μm for each field) were captured in the middle region of each part under a 40×oil objective, and the data for the labeling density (number of cells per 100 μm) were averaged for each part.

The SPSS 11.5 software package were used to perform all the statistical analyses. We used Student’s t-test and One-way ANOVA followed by Tukey’s post hoc tests to compare the differences of examined measures in the studied groups with different treatments. Normality for the examined measures was tested before statistical analysis. The data were presented as the mean ± SEM. Differences were considered nonsignificant (ns, *p* > 0.05) or significant at various levels (*p < 0.05, **p < 0.01 and ***p < 0.001).

## Supplementary information

Supplementary Dataset 2
