# Supplementary material for: Transcriptomic analysis of mouse cochleae suffering from gentamicin damage reveals the signalling pathways involved in hair cell regeneration
Source: Sci Rep. 2019 Jul 19;9:10494. doi: 10.1038/s41598-019-47051-5 (PMC6642124; doi:10.1038/s41598-019-47051-5)
Supplement: Supplementary file 1 — Supplementary Dataset 2 [file 41598_2019_47051_MOESM1_ESM.zip › Title page for Supplementary Materials.docx]

**Supplementary Materials**

**Title:** Transcriptomic analysis of mouse cochleae suffering from gentamicin damage reveals the signalling pathways involved in hair cell regeneration

**Authors:**  Huanju Bai, Lingling Jiang, Xi Wang, Xue Gao, Jie Bing, Chao Xi, Weiqian Wang, Meiguang Zhang, Xinwen Zhang, Zhongming Han, Jincao Xu and Shaoju Zeng

**Contents:**

1. STable 1(in Word format)

1. Dataset S1 (in Excel format)
