# Supplementary material for: Transcriptomic analysis of mouse cochleae suffering from gentamicin damage reveals the signalling pathways involved in hair cell regeneration
Source: Sci Rep. 2019 Jul 19;9:10494. doi: 10.1038/s41598-019-47051-5 (PMC6642124; doi:10.1038/s41598-019-47051-5)
Supplement: Supplementary file 1 — Supplementary Dataset 2 [file 41598_2019_47051_MOESM1_ESM.zip › S Table 1.doc]

**Supplementary Materials**

**Title:** Transcriptomic analysis of mouse cochleae suffering from gentamicin damage reveals the signalling pathways involved in hair cell regeneration

**Authors:**  Huanju Bai, Lingling Jiang, Xi Wang, Xue Gao, Jie Bing, Chao Xi, Weiqian Wang, Meiguang Zhang, Xinwen Zhang, Zhongming Han, Jincao Xu and Shaoju Zeng

**Table 1. Differentially expressed genes in Notch, MAPK, Wnt, TGF-β, Shh, Cell cycle, Jak-Stat signaling pathways in the mouse cochlea after gentamicin-induced damage**

| **Gene ID** | **Name** | **log_2_ (Fold Change)** | **up/down regulation** | **FDR** |
| --- | --- | --- | --- | --- |
| **Notch Signaling Pathway** | |  |  |  |
| ENSMUSG00000003436 | *DLL3* (delta-like protein 3 precursor) | -2.0867 | down | 8.88E-08 |
| ENSMUSG00000029603 | *Deltex 1* (E3 ubiquitin-protein ligase DTX1) | -1.7469 | down | 1.09E-08 |
| ENSMUSG00000048001 | *HES5* (transcription factor HES-5) | -1.7373 | down | 1.36E-07 |
| ENSMUSG00000002799 | *Jag2* (protein jagged-2 precursor) | -1.4810 | down | 3.92E-07 |
| ENSMUSG00000026923 | *Notch1* | -1.1354 | down | 0.0009 |
| ENSMUSG00000018169 | *LFNG* (beta-1,3-N-acetylglucosaminyltransferase manic fringe) | -1.0497 | down | 0.0078 |
| ENSMUSG00000049502 | *Deltex 3l* (E3 ubiquitin-protein ligase DTX3L) | 1.5683 | up | 1.64E-05 |
| **MAPK Signaling Pathway** | |  |  |  |
| ENSMUSG00000019146 | *CACNG2* (voltage-dependent calcium channel gamma-2 subunit) | -4.0696 | down | 0 |
| ENSMUSG00000018411 | *Tau* (microtubule-associated protein tau) | -3.9241 | down | 0 |
| ENSMUSG00000056553 | *PTPRN2* (receptor-type tyrosine-protein phosphatase N2 precursor) | -3.5487 | down | 0 |
| ENSMUSG00000030223 | *PTPRO* (receptor-type tyrosine-protein phosphatase O isoform 1 precursor) | -3.3869 | down | 2.00E-15 |
| ENSMUSG00000020151 | *PTPRR* (receptor-type tyrosine-protein phosphatase R isoform a precursor) | -3.1794 | down | 1.40E-05 |
| ENSMUSG00000022619 | *JIP2* (C-Jun-amino-terminal kinase-interacting protein 2) | -2.8486 | down | 0 |
| ENSMUSG00000032356 | *RasGRF1* (ras-specific guanine nucleotide-releasing factor 1 isoform 1) | -2.7433 | down | 0 |
| ENSMUSG00000020154 | *PTPRB* (receptor-type tyrosine-protein phosphatase beta precursor) | -2.6810 | down | 3.94E-07 |
| ENSMUSG00000040373 | *CACNG5* (voltage-dependent calcium channel gamma-5 subunit) | -2.4559 | down | 8.40E-11 |
| ENSMUSG00000021991 | *CACNA2D3* (voltage-dependent calcium channel subunit alpha-2/delta-3 precursor) | -2.4523 | down | 3.76E-14 |
| ENSMUSG00000004113 | *CACNA1B* (voltage-dependent N-type calcium channel subunit alpha-1B isoform 1) | -2.4475 | down | 9.71E-12 |
| ENSMUSG00000032946 | *RasGRP2* (RAS guanyl-releasing protein 2) | -2.4368 | down | 3.69E-06 |
| ENSMUSG00000022523 | *FGF12* (fibroblast growth factor 12) | -2.4093 | down | 4.45E-13 |
| ENSMUSG00000031074 | *FGF3* (fibroblast growth factor 3 precursor) | -2.2136 | down | 1.60E-07 |
| ENSMUSG00000017412 | *CACNB4* (voltage-dependent L-type calcium channel subunit beta-4 isoform a) | -2.0906 | down | 2.29E-06 |
| ENSMUSG00000004110 | *CACNA1E* (voltage-dependent R-type calcium channel subunit alpha-1E) | -2.0799 | down | 2.08E-13 |
| ENSMUSG00000046709 | *MAPK10* (mitogen activated protein kinase 10) | -2.0689 | down | 8.23E-09 |
| ENSMUSG00000025219 | *FGF8* (fibroblast growth factor 8 isoform 1 precursor) | -1.9729 | down | 0.0002 |
| ENSMUSG00000030854 | *PTPN5* (tyrosine-protein phosphatase non-receptor type 5) | -1.9146 | down | 9.90E-10 |
| ENSMUSG00000034656 | *CACNA1A* (voltage-dependent P/Q-type calcium channel subunit alpha-1A) | -1.8046 | down | 8.16E-08 |
| ENSMUSG00000047632 | *FGFBP3* (fibroblast growth factor-binding protein 3 precursor) | -1.7567 | down | 1.43E-07 |
| ENSMUSG00000057914 | *CACNB2* (voltage-dependent L-type calcium channel subunit beta-2 isoform 1) | -1.5590 | down | 1.21E-05 |
| ENSMUSG00000053141 | *PTPRT* (Receptor-type tyrosine-protein phosphatase T) | -1.4077 | down | 1.71E-05 |
| ENSMUSG00000052889 | *PKCB* (protein kinase C beta type) | -1.3821 | down | 1.44E-05 |
| ENSMUSG00000024112 | *CACNA1H* (voltage-dependent T-type calcium channel subunit alpha-1H isoform 1) | -1.3366 | down | 1.47E-05 |
| ENSMUSG00000021974 | *FGF9* (fibroblast growth factor 9 precursor) | -1.3342 | down | 0.0002 |
| ENSMUSG00000038042 | *PTPDC1* (protein tyrosine phosphatase domain-containing protein 1) | -1.2925 | down | 4.93E-05 |
| ENSMUSG00000023266 | *FRS3* (fibroblast growth factor receptor substrate 3) | -1.2810 | down | 0.0004 |
| ENSMUSG00000068748 | *PTPRZ1* (receptor-type tyrosine-protein phosphatase zeta precursor) | -1.2720 | down | 5.50E-05 |
| ENSMUSG00000010066 | *CACNA2D2* (voltage-dependent calcium channel subunit alpha-2/delta-2 isoform 1 precursor) | -1.2121 | down | 0.0008 |
| ENSMUSG00000028832 | STMN1 (stathmin) | -1.1516 | down | 0.0005 |
| ENSMUSG00000015968 | *CACNA1D* (voltage-dependent L-type calcium channel subunit alpha-1D isoform 1) | -1.1466 | down | 0.0007 |
| ENSMUSG00000027164 | *TRAF6* (TNF receptor-associated factor 6) | -1.0033 | down | 0.0067 |
| ENSMUSG00000028362 | *TNFSF8* (tumor necrosis factor ligand superfamily member 8) | 7.9021 | up | 0 |
| ENSMUSG00000027859 | *NFG* (nerve growth factor) | 5.3564 | up | 4.47E-14 |
| ENSMUSG00000063727 | *TNFRSF11B* (tumor necrosis factor receptor superfamily member 11B precursor ) | 4.8026 | up | 0 |
| ENSMUSG00000051439 | *CD14* (monocyte differentiation antigen CD14 precursor) | 4.7710 | up | 0 |
| ENSMUSG00000027399 | *IL1A* (interleukin-1 alpha precursor) | 4.6273 | up | 4.49E-12 |
| ENSMUSG00000027398 | *IL1B* (interleukin-1 beta precursor ) | 3.8819 | up | 1.21E-06 |
| ENSMUSG00000029337 | *FGF5* (fibroblast growth factor 5 isoform 1 precursor) | 3.7058 | up | 4.03E-09 |
| ENSMUSG00000045362 | *TNFRSF26* (tumor necrosis factor receptor superfamily member 26 precursor) | 3.5840 | up | 5.53E-05 |
| ENSMUSG00000024778 | *FAS* (tumor necrosis factor receptor superfamily member 6 isoform 1 precursor) | 3.3952 | up | 3.10E-10 |
| ENSMUSG00000024401 | *TNF* (tumor necrosis factor isoform 1) | 3.1753 | up | 1.94E-08 |
| ENSMUSG00000023905 | *TNFRSF12A* (tumor necrosis factor receptor superfamily member 12A isoform 1 precursor ) | 2.8494 | up | 0 |
| ENSMUSG00000070719 | *PLA2G4D* (cytosolic phospholipase A2 delta) | 2.5593 | up | 2.66E-06 |
| ENSMUSG00000002603 | *TGFB1* (transforming growth factor beta-1 precursor) | 2.4921 | up | 0 |
| ENSMUSG00000026395 | *PTPRC* (receptor-type tyrosine-protein phosphatase C isoform 1 precursor) | 2.1833 | up | 5.17E-06 |
| Mouse_newGene_12 | *PTPRV* (receptor-type tyrosine-protein phosphatase V precursor) | 2.1790 | up | 0.0026 |
| ENSMUSG00000028599 | *TNFRSF1B* (tumor necrosis factor receptor superfamily member 1B precursor) | 2.1516 | up | 8.94E-08 |
| ENSMUSG00000027347 | *RasGRP1* (RAS guanyl-releasing protein 1) | 2.0997 | up | 4.66E-12 |
| ENSMUSG00000074121 | *NT4* (neurotrophin-4 precursor) | 2.0807 | up | 3.61E-07 |
| ENSMUSG00000021250 | *FOS* (proto-oncogene c-Fos) | 2.0703 | up | 1.52E-11 |
| ENSMUSG00000026072 | *IL1R1* (interleukin-1 receptor type 1 isoform 1 precursor) | 1.9788 | up | 1.14E-09 |
| ENSMUSG00000036390 | *GADD45A* (growth arrest and DNA damage-inducible protein GADD45 alpha) | 1.9461 | up | 1.24E-10 |
| ENSMUSG00000031506 | *PTPN7* (tyrosine-protein phosphatase non-receptor type 7) | 1.9150 | up | 0.0071 |
| ENSMUSG00000037613 | *TNFRSF23* (tumor necrosis factor receptor superfamily member 23 precursor) | 1.9111 | up | 2.52E-06 |
| ENSMUSG00000070942 | *IL1RL2* (interleukin-1 receptor-like 2 precursor) | 1.8637 | up | 1.85E-07 |
| ENSMUSG00000056220 | *PLA2G4A* (cytosolic phospholipase A2) | 1.7202 | up | 5.07E-09 |
| ENSMUSG00000025225 | *NFκB* (nuclear factor NF-kappa-B p100 subunit isoform a) | 1.6966 | up | 4.27E-09 |
| ENSMUSG00000015312 | *GADD45B* (growth arrest and DNA damage-inducible protein GADD45 beta) | 1.6937 | up | 2.26E-08 |
| ENSMUSG00000024235 | *MAP3K8* (mitogen-activated protein kinase kinase kinase 8) | 1.5441 | up | 0.0003 |
| ENSMUSG00000030341 | *TNFRSF1A* (tumor necrosis factor receptor superfamily member 1A precursor) | 1.5376 | up | 8.79E-08 |
| ENSMUSG00000035678 | *TNFSF9* (tumor necrosis factor ligand superfamily member 9) | 1.4788 | up | 0.0085 |
| ENSMUSG00000055254 | *NTRK2* (neurotrophic tyrosine kinase receptor type 2) | 1.4655 | up | 5.04E-07 |
| ENSMUSG00000071369 | *MAP3K5* (mitogen-activated protein kinase kinase kinase 5) | 1.3670 | up | 2.97E-05 |
| ENSMUSG00000052684 | *JUN* (transcription factor AP-1) | 1.3528 | up | 6.82E-06 |
| ENSMUSG00000022074 | *TNFRSF10B* (tumor necrosis factor receptor superfamily member 10B precursor) | 1.2730 | up | 0.0008 |
| ENSMUSG00000025408 | *DDIT3* (DNA-damage inducible transcript 3) | 1.2463 | up | 0.0003 |
| ENSMUSG00000031530 | *DUSP4* (dual specificity protein phosphatase 4) | 1.2418 | up | 0.0001 |
| ENSMUSG00000032577 | *MAPKAPK3* (MAP kinase-activated protein kinase 3) | 1.0790 | up | 0.0024 |
| **Wnt Signaling Pathway** | |  |  |  |
| ENSMUSG00000018822 | *SFRP5* (secreted frizzled-related protein 5 precursor) | -4.5435 | down | 0 |
| ENSMUSG00000068079 | *TCF15* (transcription factor 15) | -3.6426 | down | 2.46E-05 |
| ENSMUSG00000000125 | *WNT3* (proto-oncogene Wnt-3 precursor) | -3.0407 | down | 2.10E-07 |
| ENSMUSG00000046709 | *MAPK10* (mitogen activated protein kinase 10) | -2.0689 | down | 8.23E-09 |
| ENSMUSG00000057897 | *CAMK2B* (calcium/calmodulin-dependent protein kinase II, beta, isoform CRA_b) | -1.9107 | down | 8.69E-11 |
| ENSMUSG00000051177 | *PLCB1* (1-phosphatidylinositol 4,5-bisphosphate phosphodiesterase beta-1 isoform 1) | -1.4127 | down | 0.0005 |
| ENSMUSG00000052889 | *PKCB* (protein kinase C beta type) | -1.3821 | down | 1.44E-05 |
| ENSMUSG00000020135 | *APC2* (adenomatous polyposis coli protein 2) | -1.3466 | down | 8.40E-06 |
| ENSMUSG00000055799 | *TCF7L* (transcription factor 7-like 1 isoform 1) | -1.2146 | down | 0.0001 |
| ENSMUSG00000021319 | *SFRP4* (secreted frizzled-related sequence protein 4 precursor) | 5.7144 | up | 0 |
| ENSMUSG00000026167 | *WNT10A* (protein Wnt-10a precursor) | 4.8961 | up | 3.10E-10 |
| ENSMUSG00000036309 | *SKP1* (S-phase kinase-associated protein 1) | 1.4427 | up | 1.81E-06 |
| ENSMUSG00000034739 | *MFRP* (membrane frizzled-related protein isoform 1) | 1.3579 | up | 2.99E-05 |
| ENSMUSG00000052684 | *JUN* (transcription factor AP-1) | 1.3528 | up | 6.82E-06 |
| ENSMUSG00000030170 | *WNT5B* (protein Wnt-5b) | 1.1260 | up | 0.0031 |
| ENSMUSG00000020218 | *WIF1* (wnt inhibitory factor 1 precursor) | 1.0794 | up | 0.0017 |
| **TGF-β Signaling Pathway** | |  |  |  |
| ENSMUSG00000061393 | *ACVR2B* (activin receptor type-2B precursor) | -1.4036 | down | 4.52E-06 |
| ENSMUSG00000027796 | *SMAD9* (Mothers against decapentaplegic homolog 9) | -1.1673 | down | 0.0035 |
| ENSMUSG00000052155 | *ACVR2A* (activin receptor type-2A precursor) | -1.1337 | down | 0.0007 |
| ENSMUSG00000073802 | *CDKN2B* (Cyclin-dependent kinase 4 inhibitor B) | 4.0501 | up | 0 |
| ENSMUSG00000002603 | *TGFB1* (transforming growth factor beta-1 precursor) | 2.4921 | up | 0 |
| ENSMUSG00000039004 | *BMP6* (bone morphogenetic protein 6 precursor) | 2.4182 | up | 0 |
| ENSMUSG00000040152 | *THBS1* (thrombospondin-1 precursor) | 1.6733 | up | 2.52E-09 |
| ENSMUSG00000021943 | *GDF10* ( growth differentiation factor 10) | 1.6149 | up | 1.93E-08 |
| ENSMUSG00000036309 | *SKP1* (S-phase kinase-associated protein 1) | 1.4427 | up | 1.81E-06 |
| ENSMUSG00000032179 | *BMP5* (bone morphogenetic protein 5 precursor) | 1.3647 | up | 8.88E-06 |
| ENSMUSG00000051279 | *GDF6* (growth/differentiation factor 6 precursor) | 1.3589 | up | 0.0004 |
| **Cell cycle** | | | | |
| ENSMUSG00000037664 | *CDKN1C* (cyclin-dependent kinase inhibitor 1C isoform 1) | -1.8106 | down | 5.19E-11 |
| ENSMUSG00000032411 | *TFDP2* (Transcription factor Dp-2) | -1.2608 | down | 6.72E-05 |
| ENSMUSG00000047281 | *Sfn* (14-3-3 protein sigma) | 4.1717 | up | 0 |
| ENSMUSG00000073802 | *CDKN2B* (Cyclin-dependent kinase 4 inhibitor B) | 4.0501 | up | 0 |
| ENSMUSG00000044303 | *CDKN2A* (cyclin-dependent kinase inhibitor 2A, isoform 3 isoform 1) | 3.5658 | up | 4.38E-08 |
| ENSMUSG00000002603 | *TGFB1* (transforming growth factor beta-1 precursor) | 2.4921 | up | 0 |
| ENSMUSG00000036390 | *GADD45A* (growth arrest and DNA damage-inducible protein GADD45 alpha) | 1.9461 | up | 1.24E-10 |
| ENSMUSG00000015312 | *GADD45B* (growth arrest and DNA damage-inducible protein GADD45 beta) | 1.6937 | up | 2.26E-08 |
| ENSMUSG00000027379 | *BUB1* (mitotic checkpoint serine/threonine-protein kinase BUB1 isoform 1) | 1.2969 | up | 0.0004 |
| ENSMUSG00000027715 | *CCNA2* (Cyclin-A2) | 1.1159 | up | 0.0016 |
| ENSMUSG00000030867 | *PLK1* (serine/threonine-protein kinase PLK1) | 1.0638 | up | 0.0053 |
| **Hedgehog Signaling Pathway** | | | | |
| ENSMUSG00000000125 | *WNT3* (proto-oncogene Wnt-3 precursor) | -3.0407 | down | 2.10E-07 |
| ENSMUSG00000023000 | *DHH* (desert hedgehog protein precursor) | -2.4412 | down | 1.89E-15 |
| ENSMUSG00000046709 | *MAPK10* (mitogen activated protein kinase 10) | -2.0689 | down | 8.23E-09 |
| ENSMUSG00000064325 | *HHIP* (hedgehog-interacting protein precursor) | -1.9960 | down | 4.12E-08 |
| ENSMUSG00000030170 | *WNT5B* (protein Wnt-5b) | 1.1260 | up | 0.0031 |
| **Jak-Stat Signaling Pathway** | | | | |
| ENSMUSG00000031304 | *IL2RG* (interleukin 2 receptor, gamma chain) | 2.9392 | up | 1.01E-06 |
| ENSMUSG00000053113 | *SOCS3* (suppressor of cytokine signaling 3) | 2.4989 | up | 0 |
| ENSMUSG00000004040 | *STAT3* (signal transducer and activator of transcription 3 isoform 1) | 1.9360 | up | 1.39E-12 |
| ENSMUSG00000026104 | *STAT1* (Signal transducer and activator of transcription 1) | 1.7753 | up | 1.14E-09 |
| ENSMUSG00000031805 | *JAK3* (Janus kinase 3) | 1.5545 | up | 4.04E-07 |
| ENSMUSG00000002147 | *STAT6* (signal transducer and transcription activator 6) | 1.4430 | up | 1.64E-06 |
| ENSMUSG00000004043 | *STAT5A* (signal transducer and activator of transcription 5A) | 1.3845 | up | 2.02E-05 |
| ENSMUSG00000038037 | *SOCS1* (suppressor of cytokine signaling 1) | 1.1066 | up | 0.0053 |
| ENSMUSG00000040033 | *STAT2* (signal transducer and activator of transcription 2) | 1.0395 | up | 0.0044 |
| ENSMUSG00000037104 | *SOCS5* (suppressor of cytokine signaling 5) | 0.9874 | up | 0.0084 |
